# Prevention and treatment of ischaemic and haemorrhagic stroke in people with diabetes mellitus: a focus on glucose control and comorbidities

**DOI:** 10.1007/s00125-024-06146-z

**Published:** 2024-04-16

**Authors:** Simona Sacco, Matteo Foschi, Raffaele Ornello, Federico De Santis, Riccardo Pofi, Michele Romoli

**Affiliations:** 1 Department of Biotechnological and Applied Clinical Sciences, University of L’Aquila, L’Aquila, Italy; 2 Oxford Centre for Diabetes, Endocrinology and Metabolism, NIHR Oxford Biomedical Research Centre, Churchill Hospital, University of Oxford, Oxford, UK; 3 Neurology and Stroke Unit, Department of Neuroscience, Bufalini Hospital, Cesena, Italy

**Keywords:** Antithrombotics, Chronic kidney diseases, Diabetes, Endothelial damage, Hyperglycaemia, Review, Small vessel disease, Stroke

## Abstract

Diabetes mellitus is a significant risk factor for both ischaemic and haemorrhagic stroke, affecting up to a third of individuals with cerebrovascular diseases. Beyond being a risk factor for stroke, diabetes and hyperglycaemia have a negative impact on outcomes after ischaemic and haemorrhagic stroke. Hyperglycaemia during the acute ischaemic stroke phase is associated with a higher risk of haemorrhagic transformation and poor functional outcome, with evidence in favour of early intervention to limit and manage severe hyperglycaemia. Similarly, intensive glucose control nested in a broader bundle of care, including blood pressure, coagulation and temperature control, can provide substantial benefit for clinical outcomes after haemorrhagic stroke. As micro- and macrovascular complications are frequent in people with diabetes, cardiovascular prevention strategies also need to consider tailored treatment. In this regard, the broader availability of sodium–glucose cotransporter 2 inhibitors and glucagon-like peptide 1 receptor agonists can allow tailored treatments, particularly for those with heart failure and chronic kidney disease as comorbidities. Here, we review the main concepts of hyperacute stroke management and CVD prevention among people with diabetes, capitalising on results from large studies and RCTs to inform clinicians on preferred treatments.

**Graphical Abstract:**

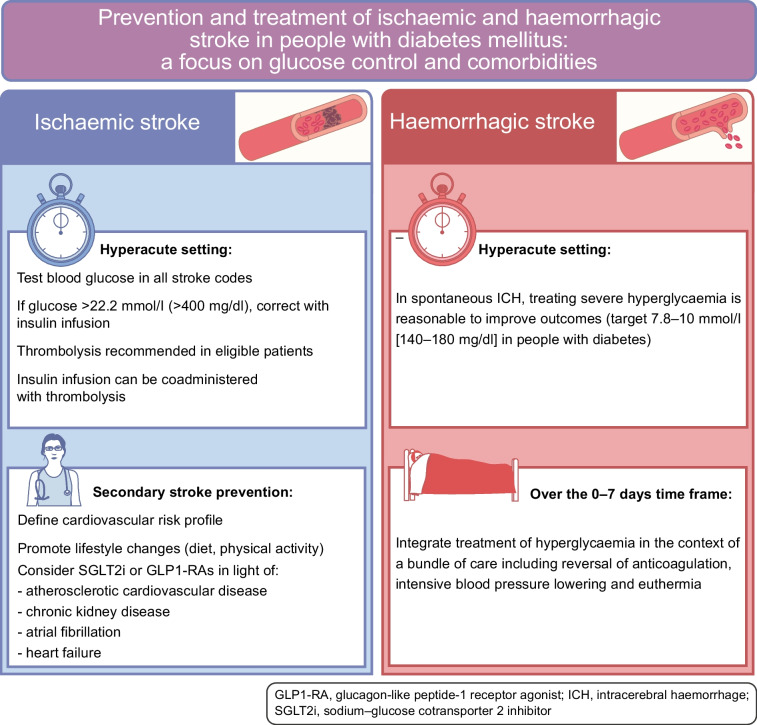

**Supplementary Information:**

The online version contains a slideset of the figures for download available at 10.1007/s00125-024-06146-z.

## Introduction

Diabetes mellitus is a significant global health concern, with CVD being the most common cause of death among adults with diabetes. Individuals with type 2 diabetes have a risk of death from cardiovascular (CV) causes that is two to six times higher than that among individuals without diabetes [1, 2].

Stroke is a leading cause of serious long-term disability and death, with the incidence of stroke rapidly increasing with age and doubling for each decade after age 55 years [3]. People with type 2 diabetes have approximately twice the risk of stroke as people without diabetes, with up to 40% of stroke cases potentially attributable to the effects of diabetes alone or in combination with hypertension [4]; the prevention of cerebrovascular disease (CVD) is therefore critical among this population. Beyond type 2 diabetes, hyperglycaemia is also a strong risk factor for poor outcome after acute stroke in both people with diabetes and those without diabetes [5]. Stress hyperglycaemia, an acute and transient rise in blood glucose levels triggered by stressful events, including stroke [6], should be clearly distinguished from chronic hyperglycaemia, a long-term consequence of diabetes that can lead to vascular disease. In the setting of stroke, the management of hyperglycaemia is critical in the acute phase of the disease [5, 7], while the management of chronic hyperglycaemia is important for primary and secondary prevention in people with diabetes [8].

This review summarises the evidence on the associations between type 2 diabetes and CVD, focusing on the management and preventive treatment of ischaemic and haemorrhagic stroke in people with diabetes.

## Epidemiology of diabetes and cerebrovascular events

Diabetes remains a substantial public health issue, with type 2 diabetes accounting for the majority of diabetes cases [9]. Type 2 diabetes is largely preventable and, in some cases, potentially reversible if identified and managed early in the disease course. Substantial evidence indicates that diabetes prevalence is increasing worldwide, primarily due to a rise in obesity, with more than 50% of cases of diabetes attributable to high BMI [9]. Prevention and early treatment of diabetes is critical to limit delayed micro- and macrovascular complications.

One in four people with acute stroke has diabetes, and both diabetes and high blood glucose confer a consistent relative increase in risk of in-hospital mortality and poor functional outcome after stroke [10]. Data from the Northern Manhattan Study highlighted a clear temporal relationship between stroke and diabetes, with a 3% annual increase in risk of stroke per year of diabetes duration, and a threefold increase in risk of stroke in those with diabetes compared with the population without diabetes [11]. Summary estimates from a large meta-analysis of 102 prospective studies highlighted that diabetes confers a 2.3-fold increased risk of ischaemic stroke and a 1.6-fold increased risk of haemorrhagic stroke, suggesting that diabetes is among the main drivers of vascular events overall [12]. For ischaemic stroke, according to estimates from population studies, up to 40% of cases may be attributable to diabetes [4]. Similarly, with regard to haemorrhagic stroke, up to 26% of cases may be attributable to diabetes, therefore highlighting a CV burden that has to be managed [12].

Notably, both type 1 and type 2 diabetes are associated with an increased risk of stroke compared with the absence of diabetes [13]. Prediabetes is also associated with an increased risk of stroke [14]. The present review is focused on type 2 diabetes, for which treatment approaches and glycaemic control can impact the risk and development of micro- and macrovascular disease.

## Pathological mechanisms in CV diseases associated with diabetes

### Arterial stiffness

Arterial stiffness develops in relation to microvascular dysfunction in people with diabetes [15, 16], involves all territories, including cerebral vessels, and represents an independent predictive factor for stroke and cerebrovascular events [15, 16]. Arterial stiffness is associated with all-cause mortality and CV events, with a 1 unit lower carotid distension index associated with a 4% increase in risk of cerebrovascular events [15, 16]. Arterial stiffness seems to develop early in the course of the metabolic syndrome, also preceding and representing a risk factor for diabetes over time, and correlating with fasting blood glucose even in people without diabetes [17]. People with hypertension and arterial stiffness have a 2.5-fold increased risk of developing diabetes, suggesting that a metabolic process leading to diabetes and vascular changes happens well in advance of diabetes onset [18]. Indeed, more than a third of people with diabetes present with arterial stiffness at the time of diabetes diagnosis, highlighting the need to target arterial stiffness early to avoid vascular complications [19]. To this extent, deteriorating glucose tolerance is associated with increased central and peripheral arterial stiffness, highlighting that there may be room for early management to limit complications and lower the risk of CV events [20].

### Small vessel disease

Both people with diabetes and those with prediabetes have been shown to suffer from cerebral small vessel disease (SVD) [2, 21]. Cerebral microvascular dysfunction is hypothesised to be a major contributor to the development of SVD, and may be sustained by several factors that are common among people with diabetes, including hyperglycaemia, obesity, insulin resistance and hypertension [2]. As arterial stiffness develops early in the course of diabetes, this may well add to the development of cerebral SVD, exposing small-calibre vessels to abnormal pulsatility [2, 21]. Microvascular dysfunction can further be promoted by microalbuminuria, with increases in levels of endothelial growth factors and vascular cell adhesion molecule potentially promoting the development of SVD in people with diabetes [2, 21]. Given the progressive and subtle course of microvascular impairment, people with diabetes can be investigated for signs of microvascular dysfunction before clinical manifestations. Cerebral SVD is associated with microvascular damage to the retina and the progression of diabetic retinopathy [22, 23], but also with cognitive function. In the Age, Gene/Environment Susceptibility (AGES)–Reykjavik study, compared with people without diabetes, people with diabetes had lower scores on cognitive testing, particularly regarding executive function [24]. This association was mediated by brain markers of cerebral SVD, pointing to a peculiar contribution of brain microvascular dysfunction to cognitive performance [21, 24].

### Diabetic cardiomyopathy

In addition to the commonly recognised microvascular complications of diabetes, such as nephropathy, retinopathy and neuropathy [25], a distinct pathological entity known as diabetic cardiomyopathy has been increasingly acknowledged [26]. Distinct from coronary artery disease, hypertension or valvular heart disease, diabetic cardiomyopathy represents a unique cluster of structural and functional cardiac alterations in people with diabetes. Diabetic cardiomyopathy is progressive and encompasses a continuum that has been well defined by the American College of Cardiology Foundation/American Heart Association [27].

The initial asymptomatic stage of diabetic cardiomyopathy is characterised by isolated increased myocardial stiffness, leading to reduced left ventricular (LV) compliance, impaired early diastolic filling and prolonged isovolumetric relaxation, which all contribute to elevated LV end-diastolic pressure [26]. This stage eventually progresses to worsening LV hypertrophy and diastolic dysfunction, culminating in heart failure with preserved ejection fraction (HFpEF). HFpEF seems more common among people with diabetes than among the general population, probably due to endothelial dysfunction and insulin resistance within myocytes fostered by the accumulation of lipids within non-adipose tissue [28, 29].

As well as HFpEF, people with diabetes can also develop heart failure with reduced ejection fraction (HFrEF), a condition characterised by prolonged pre-ejection periods, shortened ejection times and increased filling resistance and pressures [30, 31]. In people with HFrEF, changes in glycaemic control are common, and diabetes is associated with a higher risk of adverse CV outcomes compared with the absence of diabetes [31]. Despite growing recognition of the cardiac risks associated with diabetes, the impact of glycaemic control on the progression of diabetic cardiomyopathy remains uncertain. Observational data suggest that strict glycaemic management does not significantly alter the course of cardiac disease progression [32]. However, hyperglycaemia is a critical factor in activating inflammatory and profibrotic pathways within the myocardium and is associated with an increased risk of heart failure progression, evidenced by an 8% elevation in risk for each 1% increase in HbA_1c_ [33]. The pathophysiology of diabetic cardiomyopathy encompasses a broad spectrum of changes, including alterations in the cardiac extracellular matrix, viscoelastic properties, contraction dynamics, cardiomyocyte signalling pathways and proinflammatory responses [30]. Clinically, diabetic cardiomyopathy is significant due to its association with an increased incidence of acute coronary syndrome and the development of asymptomatic ischaemic scars, which occur regardless of effective metabolic control. Therefore, early recognition is crucial to enable glycaemic control to be restored before the cascade leading to diabetic cardiomyopathy is activated.

### Chronic kidney disease

Chronic kidney disease (CKD) occurs in 10–90% of people with diabetes, with the overall burden likely to increase over time [34]. The development of CKD in people with diabetes intensifies their morbidity and increases their risk of mortality, particularly from CVD-related death [35]. Individuals with both CKD and diabetes have a higher risk of stroke than those with diabetes alone. The Kidney Disease: Improving Global Outcomes (KDIGO) 2022 Clinical Practice Guideline for Diabetes Management in Chronic Kidney Disease emphasises the increased risk of incident stroke in adults with type 2 diabetes and CKD, which is particularly associated with higher albuminuria levels, decreased eGFR and worsening CKD stage [36]. CKD promotes the progression of arteriolosclerosis and endothelial dysfunction and increases the burden of SVD and vascular calcifications, further increasing the risk of stroke [37]. CKD also negatively impacts arterial stiffness. To this extent, the strain vessel hypothesis suggests that both juxtamedullary afferent arterioles and cerebral perforating arteries are exposed to high blood pressure and have to maintain large pressure gradients, rendering them extremely susceptible to hypertensive injury [37]. In addition, CKD progression can also contribute to oxidative stress, endothelial dysfunction and chronic inflammation, predisposing people with diabetes to a higher SVD burden, as well as higher risks of cerebrovascular events and cognitive decline [38].

## Treatment: impact of diabetes on reperfusion treatments and management of hyperglycaemia in the hyperacute stage

People with diabetes tend to have a higher prevalence of comorbid factors and vascular conditions that increase the risk of stroke [10]. As hyperacute stroke care has evolved over the last few decades, an important question is whether diabetes itself or hyperacute hyperglycaemia can have a negative impact on stroke reperfusion treatments. Here, we summarise the impact of diabetes on i.v. thrombolysis (IVT) and endovascular thrombectomy (EVT) in hyperacute ischaemic stroke, and provide a clinical overview of hyperglycaemia management in the hyperacute stroke setting. Harmful effects and molecular mechanisms of chronic hyperglycaemia and insulin resistance, inflammation, oxidative stress, advanced glycation end-products and endothelial damage are reviewed in-depth elsewhere [39–41].

### Ischaemic stroke

In ischaemic stroke, clinical impairment is due to a poorly perfused but salvageable part of the brain (penumbra) that gradually transforms into permanently damaged tissue (core) if left untreated [42]. Reperfusion therapies, which are time sensitive, can prevent such evolution. IVT can mitigate disability when administered within appropriate time and tissue-based windows [43]. Large vessel occlusion (LVO) stroke is an ischaemic stroke resulting from the occlusion of one of the main intracranial branches of the internal carotid artery, including the anterior and middle cerebral arteries in their proximal segments, or the vertebra-basilar arteries. LVO stroke accounts for more than 50% of stroke cases and can be treated with EVT [44, 45]. EVT reduces disability in people with LVO by mechanically removing blood clots using catheter angiography. EVT should ideally be performed within 6 h of an individual’s last known healthy state; however, in selected cases, based on brain perfusion imaging, it may be considered up to 24 h from ischaemic stroke onset [44, 45].

To date, recombinant tissue plasminogen activator (rt-PA) remains the only approved pharmaceutical agent for acute ischaemic stroke management. In two of the RCTs on rt-PA (European Cooperative Acute Stroke Study [ECASS-3] and the third International Stroke Trial [IST-3]), known diabetes did not modify the effect of IVT on good functional outcomes at 6 months or increase mortality rates [46, 47]. Therefore, current guidelines strongly recommend IVT in individuals with known diabetes when administrated within the appropriate time window [48]. In this regard, the substantial equipoise of tenecteplase to alteplase has been confirmed in people with stroke and diabetes [49]; therefore, ensuring that, regardless of the treatment available, diabetes should not be a contraindication to IVT [50]. Of interest, ongoing treatment with metformin was associated with a better functional outcome after 3 months in a propensity matched retrospective analysis of individuals with stroke treated with IVT in the European Thrombolysis in Ischemic Stroke Patients (TRISP) collaboration [51], potentially related to ischaemic and anaerobic metabolism pre-conditioning [52].

The impact of diabetes and hyperglycaemia on safety and functional outcome after EVT is unclear. Studies have suggested that hyperglycaemia increases the risk of poor functional outcome after EVT, especially in individuals with incomplete reperfusion. A post hoc analysis of data from the Solitaire Flow Restoration With the Intention for Thrombectomy (SWIFT) multicentre RCT showed that participants with baseline serum glucose levels >7.8 mmol/l (140 mg/dl) were at a higher risk of worse functional outcome at 3 months [53]. Data from the Multicenter Randomised Clinical Trial of Endovascular Treatment for Acute Ischemic Stroke in the Netherlands (MR CLEAN) showed no evidence for effect modification of EVT by admission serum glucose levels in participants with acute ischaemic stroke, with similar rates of symptomatic intracranial haemorrhage in people with and without diabetes [54].

#### Management of hyperglycaemia in hyperacute ischaemic stroke

**Fig. 1:**
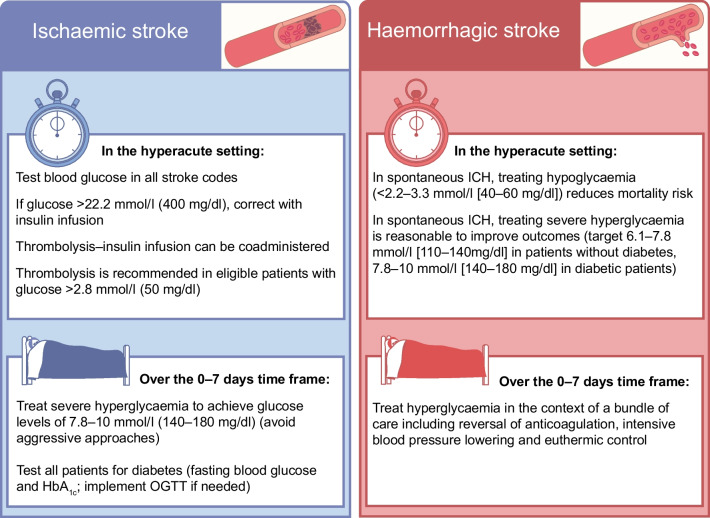
Main recommendations for the management of hyperglycaemia in the context of acute and subacute ischaemic and haemorrhagic stroke. Based on European and American guidelines for the management of ischaemic stroke [48, 55] and selected RCTs [61, 68]. This figure is available as part of a downloadable slideset

Current guidelines for acute ischaemic stroke treatment recommend the assessment of blood glucose levels before the initiation of thrombolysis in all individuals with acute ischaemic stroke [48, 55]. Such an approach is meant to ensure that all individuals receive thrombolysis for acute stroke, and in the recommended setting, as severe hyperglycaemia or hypoglycaemia can manifest with stroke-like deficit [56]. Higher blood glucose levels at admission are associated with poorer functional outcome and major bleeding in those with acute ischaemic stroke receiving thrombolysis, independently of age, coagulopathy and stroke severity [5, 7]. Indeed, an elevated serum glucose level was recognised as an independent risk factor for haemorrhagic transformation in the National Institute of Neurological Disorders and Stroke rt-PA Trial, regardless of IVT administration [57]. Individuals with blood glucose levels >22.2 mmol/l (>400 mg/dl) were not included in the ECASS-3 and IST-3 trials [46, 47], so the only available evidence comes from observational studies and registries. In the Virtual International Stroke Trials Archive (VISTA), 23 participants with blood glucose levels above the threshold level (22.2 mmol/l [400 mg/dl]) had similar outcomes to those with lower levels (although only six received thrombolysis) [58]. However, in the SITS registry, compared with participants with blood glucose levels of 4.4–6.7 mmol/l (80–120 mg/dl), people with levels of 10.1–11.1 mmol/l (181–200 mg/dl) had a 2.9-fold increased risk of symptomatic intracranial haemorrhage [59], supporting the need to treat hyperglycaemia promptly. In this regard, RCTs have investigated the potential impact of intensive blood glucose management in people with hyperglycaemia in the hyperacute ischaemic stroke setting. In the Glucose Insulin in Stroke Trial (GIST) [60], glucose–potassium–insulin (GKI) infusions aimed at maintaining euglycaemia (4–7 mmol/l [72–126 mg/dl]) were given immediately after the acute event, but no impact on 90 day mortality emerged (GKI vs control: OR 1.14, 95% CI 0.86, 1.51). As the trial was stopped early due to the low recruitment rate, and only a marginal difference in blood glucose levels was achieved between arms (0.6 mmol/l [10.8 mg/dl]), the certainty of the equivalence of treatment effect was uncertain. In the Stroke Hyperglycaemia Insulin Network Effort (SHINE) RCT, adults with hyperglycaemia and acute ischaemic stroke were randomly allocated to receive continuous i.v. insulin (intensive treatment; target blood glucose concentration 4.4–7.2 mmol/l [(80–130 mg/dl]) or s.c. insulin (standard treatment; target blood glucose concentration 4.4–9.9 mmol/l [80–179 mg/dl]) for up to 72 h. Overall, despite a mean difference of 3.4 mmol/l (61 mg/dl) in blood glucose levels across groups, intensive insulin treatment did not improve functional outcome after stroke compared with standard treatment [61]. Drawing lessons from the management of other microvascular complications, such as the exacerbation of diabetic retinopathy after aggressive insulin therapy [62], a tailored approach is advisable in addressing severe hyperglycaemia in those with acute ischaemic stroke. It is reasonable, particularly in the first 72 h, to avoid aggressive glucose-lowering treatment, while ensuring that severe hyperglycaemia is meticulously managed with the aim of balancing efficacy and safety (Fig. 1).

### Haemorrhagic stroke

Diabetes and hyperglycaemia can negatively affect the outcomes of individuals with intracerebral haemorrhage (ICH). Both conditions are associated with an increased risk of early haematoma expansion and in-hospital death and a higher burden of disability in survivors [63, 64]. In a post hoc analysis of the randomised Intensive Blood Pressure Reduction in Acute Cerebral Hemorrhage (INTERACT-2) trial, persistent (>24 h) hyperglycaemia at ICH presentation was strongly associated with poor outcome and major disability (modified Rankin Scale [mRS] ≥3), while known diabetes mostly predicted residual disability [65]. The effect of moderate (7.8–10 mmol/l [140–180 mg/dl] blood glucose) and severe (≥10 mmol/l [≥180 mg/dl] blood glucose) persistent hyperglycaemia on post-ICH outcomes was evaluated in a large cohort of individuals enrolled in the Antihypertensive Treatment of Acute Cerebral Hemorrhage 2 (ATACH-2) trial [66]. Both moderate and severe hyperglycaemia were independently associated with higher 90 day death or disability rates in people with diabetes, further supporting the impact of hyperglycaemia on ICH in the hyperacute setting [66]. Of note, intensive reduction of systolic blood pressure in individuals with ICH was associated with a lower rate of haematoma expansion in normoglycaemic individuals than in those with hyperglycaemia [66].

#### Management of hyperglycaemia in acute haemorrhagic stroke

As hyperglycaemia is also associated with poor outcomes after haemorrhagic stroke, the potential role of intensive glucose control in such populations has been tested in several trials. In the UK Glucose Insulin in Stroke Trial, 114 participants who presented within 24 h of symptom onset were randomised to receive either continuous i.v. GKI infusions for a minimum of 24 h, aiming to maintain blood glucose in the range 4–7 mmol/l (72–126 mg/dl), or 0.9% normal saline (154 mmol/l NaCl) at 100 ml/h for 24 h to maintain blood glucose levels below 17 mmol/l (306 mg/dl). There were no differences between groups in all-cause mortality and death or disability at 90 days, although the trial was underpowered to detect such differences [60]. In a systematic review of 16 RCTs involving 1248 neurocritical care participants with various conditions, including ICH, intensive glucose control had no significant effect on mortality risk compared with standard glycaemic control, but did result in fewer unfavourable neurological outcomes [67]. The Intensive Care Bundle with Blood Pressure Reduction in Acute Cerebral Haemorrhage Trial (INTERACT-3) trial focused on blood glucose control within the implementation of a care bundle protocol, which also included early intensive lowering of systolic blood pressure (<140 mmHg), antipyretic treatment (≤37.5°C) and rapid reversal of warfarin-related anticoagulation. In this context, the integration of intensive glucose control (target 6.1–7.8 mmol/l [110–140 mg/dl] in participants without diabetes and 7.8–10 mmol/l [140–180 mg/dl] in those with diabetes) with the other measures was associated with a significantly lower likelihood of poor functional outcome and mortality [68]. Of note, participants in the care bundle group had shorter hospital stays, a higher quality of life and a 4% reduction in serious adverse events compared with those in the usual care group, suggesting that implementing intensive glucose control as part of comprehensive care may indeed provide substantial benefits [68]. International guidelines on the management of patients with spontaneous ICH also recommend glucose monitoring to control and prevent hypoglycaemia, for which treatment is recommended when blood glucose levels are below 2.2–3.3 mmol/l (40–60 mg/dl) [69] (Fig. 1).

## Stroke prevention

Primary stroke prevention in people with diabetes hinges on managing modifiable risk factors through lifestyle adjustments or pharmacological or surgical interventions [2]. Several studies have shown that intensive management of multiple risk factors can significantly reduce the occurrence of first-time and recurrent strokes in individuals with diabetes [70, 71]. Accordingly, 2023 European Society of Cardiology (ESC) guidelines for the management of CV disease in people with diabetes strongly recommend smoking cessation, increasing physical activity levels and weight loss in individuals living with overweight or obesity [72]. As shown by the Swedish National Diabetes Register, which considered the relevance of five risk factors (HbA_1c_ >53 mmol/mol [7%], elevated LDL-cholesterol level, albuminuria, smoking and elevated blood pressure), a high HbA_1c_ level was the strongest predictor of stroke in people with type 2 diabetes [73].

While both acute and chronic hyperglycaemia have been linked to increased stroke risk and severity, aggressively lowering glucose levels has not proven effective in preventing major vascular events such as stroke [74]. Indeed, tight glycaemic control (HbA_1c_ <53 mmol/mol [7%]) has been associated with a reduced risk of microvascular complications, but effects on stroke are more complex [8]. A multifaceted approach to managing glucose, blood pressure and lipids, coupled with medications such as renin–angiotensin system inhibitors, statins and aspirin (where appropriate), has been shown to reduce both microvascular and macrovascular complications of diabetes, including stroke [75]. Evidence from CV outcome trials suggests that specific glucose-lowering agents, such as sodium–glucose cotransporter 2 (SGLT2) inhibitors and glucagon-like peptide-1 receptor agonists (GLP-1 RAs), can reduce the risk of major CV events in individuals with diabetes (Table 1) [76]. On the contrary, dipeptidyl peptidase-4 (DPP-4) inhibitors seem not to provide any CV advantage [77]. The impact of other glucose-lowering agents such as sulfonylureas, metformin and alpha-glucosidase inhibitors on CV outcomes remains uncertain. Similarly, basal insulin treatment has been shown to have a neutral effect on CV outcomes, including stroke [78]. Additionally, meta-analyses of randomised trials with a duration of over 12 months have indicated that most glucose-lowering therapies have no significant impact on stroke risk, except for thiazolidinediones and GLP-1 RAs [79]. GLP-1 RAs may reduce the risk of major CV events by multiple mechanisms, including a reduction in HbA_1c_, LDL-cholesterol levels, blood pressure, weight, urine albumin/creatinine ratio and high-sensitivity C-reactive protein levels [80]. Therefore, choice of primary prevention should prioritise CV risk factors to limit the risk of major CV events in people with diabetes.

**Table 1:** Interventions associated with reduction in the risk of fatal or non-fatal stroke in individuals with type 2 diabetes

Study	Design	Intervention	Risk of fatal or non-fatal stroke		
			Calculated risk	95% CI	*p* value
Steno-2 (2016) [75]	RCT	Intensive treatment of CV risk factors (stepwise implementation of behaviour modification, medications targeting hyperglycaemia, hypertension, dyslipidaemia and microalbuminuria)	HR 0.31	0.14, 0.69	0.004
GLP-1 RAs					
SUSTAIN-6 (2016) [98]	RCT	Semaglutide (weekly)	HR 0.61	0.38, 0.99	0.004
REWIND (2019) [99]	RCT	Dulaglutide	HR 0.76	0.62, 0.94	0.010
Benn (2021) [79]	Meta-analysis	Overall (class effect)	RR 0.85	0.77, 0.94	0.002
Sattar (2021) [76]	Meta-analysis	Overall (class effect)	RR 0.83	0.76, 0.92	<0.001
Thiazolidinediones					
Benn (2021) [79]	Meta-analysis	Overall (class effect)	RR 0.82	0.69, 0.98	0.025
Secondary stroke prevention					
Thiazolidinediones					
PROactive (2005) [81]	RCT	Pioglitazone	HR 0.53	0.34, 0.85	0.009
IRIS (2019) [100]	RCT	Pioglitazone	HR 0.76	0.62, 0.93	0.007

### Secondary prevention

Thiazolidinediones have been a subject of interest in the context of CV complications in individuals with diabetes. The PROspective pioglitAzone Clinical Trial In macroVascular Events (PROactive) initially demonstrated a non-significant reduction in stroke risk with pioglitazone among individuals with diabetes without prior CV events [81]. Intriguingly, a subsequent post hoc analysis highlighted that, in individuals with a previous history of stroke, pioglitazone significantly lowered the risk of recurrence, supporting a direct application in practice [82]. Data from the Insulin Resistance Intervention after Stroke (IRIS) randomised trial also highlight the potential benefit of pioglitazone in people with insulin resistance. In particular, people with insulin resistance and a recent stroke or transient ischaemic attack (TIA) showed a consistent reduction in rates of myocardial infarction with pioglitazone compared with placebo, and pioglitazone was associated with a decrease in risk of diabetes over the following 4.8 years [83]. Such an effect suggests that pioglitazone’s benefits extend beyond glycaemic control to impact inflammation, fat distribution, lipid and protein metabolism and vascular endothelial function [83]. However, the clinical application of pioglitazone requires consideration of its delayed maximal effect, which can span several weeks. Moreover, treatment-associated adverse effects, such as weight gain, increased fracture risk and fluid retention [83], are of particular concern in those with pre-existing CVD. Given these potential drawbacks, pioglitazone may not always be the optimal first-line therapy in such cases. In contrast to pioglitazone, rosiglitazone has a different risk profile. One study has linked rosiglitazone with an elevated risk of stroke, potentially attributable to its effects on increasing plasma cholesterol and triacylglycerol levels [79]. This distinction between the two drugs underscores the importance of individualised treatment strategies in the management of individuals with diabetes and CV risk factors.

### Special consideration for people with CV comorbidities

**Fig. 2:**
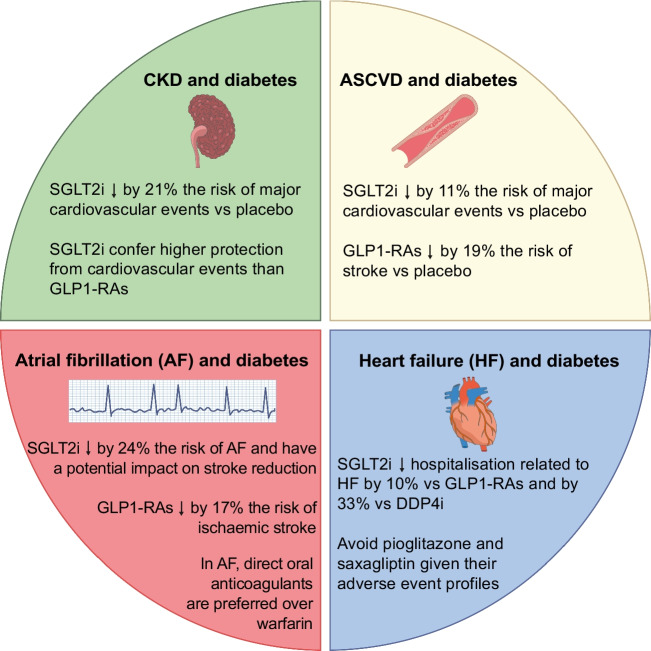
Special considerations for the management of diabetes with glucose-lowering medications in vulnerable subgroups of people with diabetes and CKD, ASCVD, atrial fibrillation or heart failure. DPP-4i, dipeptidyl peptidase 4 inhibitors; SGLT2i, sodium–glucose cotransporter 2 inhibitors. Based on results and approximated estimates in [85–95]. This figure is available as part of a downloadable slideset

Glucose-lowering medications can be prescribed with the aim of glucose control, but also with the target of improving CV outcomes in people with diabetes. Special consideration should be given to comorbidities and concurrent CV risk factors, tailoring CVD prevention based on the results of CV outcomes-based trials of glucose-lowering medications [72] (Fig. 2).

In people with diabetes and concomitant atherosclerotic cardiovascular disease (ASCVD), SGLT2 inhibitors seem to have a protective effect on the risk of stroke, myocardial infarction and CV death. In a meta-analysis of the six major trials on use of SGLT2 inhibitors in individuals with type 2 diabetes, those with diabetes and ASCVD had an 11% reduction in the HR for major CV events compared with placebo, an effect that was only marginally present in people without ASCVD [84]. Similarly, GLP1-RAs had a significant benefit for CV outcomes in people with diabetes at risk of ASCVD. In a meta-analysis including seven randomised placebo-controlled studies, stroke occurrence was reduced by 19% compared with placebo, with benefits also seen for hospitalisation, major CV events and CV death [76]. Consequently, according to ESC guidelines on the management of CVD in individuals with diabetes, SGLT2 inhibitors and GLP1-RAs are the recommended treatments in those with a high risk of ASCVD, independent of glucose control considerations and independent of background metformin use [72]. However, the currently available evidence from meta-analyses shows that the risk of stroke in people with ASCVD was specifically decreased by GLP1-RAs [76], while the effect of SGLT2 inhibitors on stroke risk seemed neutral [84]. The RCTs available also did not focus on individuals with stroke, therefore providing, at best, indirect evidence on the benefits of GLP1-RAs and SGLT2 inhibitors for secondary stroke prevention.

In people with diabetes and atrial fibrillation (AF), the impact of SGLT2 inhibitors and GLP1-RAs is to some extent more controversial. AF is relevant for stroke prevention, as it predisposes to cardioembolism, with the risk mitigated by anticoagulants [85, 86]. Nevertheless, individuals with AF have an increased residual risk for ischaemic stroke, even with optimal anticoagulation [86]. People with diabetes have a 35% higher risk of AF than the general population [87]. Treatment of AF with anticoagulants does not differ between people with diabetes and those without diabetes [88]. In particular, as SGLT2 inhibitors and GLP1-RAs do not interact with direct oral anticoagulants [89], clinicians should focus on AF prevention and appropriate dosage recommendations in light of CVD. SGLT2 inhibitors were associated with a 24% reduction in the odds of developing AF in a meta-analysis including 13 placebo-controlled trials [90], although this effect did not seem to translate into a significant reduction in stroke rates [91]. However, data from large observational studies support the overall effect of SGLT2 inhibitors, with a reduction in risk of stroke of 20% compared with non-users even after adjustment for CV risk factors [92].

In people with heart failure (HF) and diabetes, SGLT2 inhibitors seem to have a critical advantage in terms of the prevention of deterioration of cardiac function and hospitalisation compared with other treatments. In a large observational study, compared with GLP-1RAs, SGLT2 inhibitor use was associated with a significantly lower risk of hospitalisation for HF, ranging from 14% in people with HFrEF to 11% in those with HFpEF [93]. SGLT2 inhibitors were also superior to DPP-4 inhibitors in terms of hospitalisation for HF, with a 33% reduction in rate, but also in terms of myocardial infarction and stroke, with a 14% reduction in rates [93]. Therefore, SGLT2 inhibitors are recommended as first-choice, independently of glucose control or other concomitant glucose-lowering treatment, in individuals with diabetes and HF, to reduce the rate of HF-related events. Given their increased risk of HF, pioglitazone and saxagliptin are not recommended in this subgroup of patients [72].

CKD often accompanies diabetes and therefore special consideration should be given to people with decreasing renal function receiving glucose-lowering agents. In a landmark meta-analysis of 13 trials with a total of 90,409 participants, SGLT2 inhibitors reduced the risk of kidney disease progression by 37% compared with placebo, therefore emerging as a potential treatment to modify the course of the disease [94]. In addition, the positive effect extended to kidney injury, which was reduced by 21%, and was independent of the presumed primary kidney disease, implying a broad generalisability [94]. In a network meta-analysis comparing SGLT2 inhibitors with GLP1-RAs in participants with diabetes and CKD, SGLT2 inhibitors had a lower risk of major CV events, supporting their implementation as first-choice therapy in people with diabetes and CKD for CV prevention purposes [95].

## Neurologist and diabetes specialist approach to people with stroke and diabetes

**Fig. 3:**
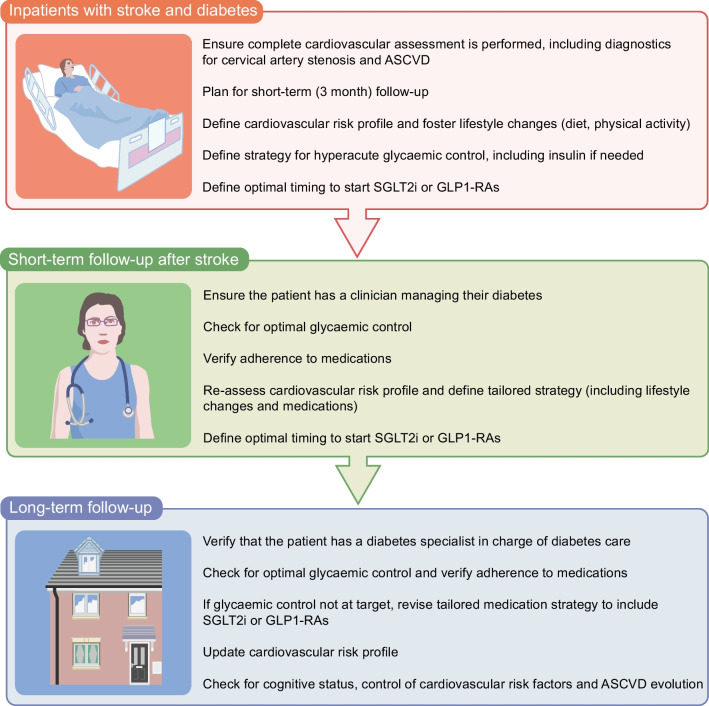
Pragmatic multidisciplinary approach to the care of people with ischaemic or haemorrhagic stroke and diabetes. This figure is available as part of a downloadable slideset

The involvement of neurologists in the care of individuals with stroke and diabetes, as well as neurological examination during diabetes specialist care, provide critical opportunities to improve the care of individuals with diabetes (Fig. 3) [96]. A collaborative approach between neurologists and endocrinologists has the common aim of providing the most appropriate treatment, from hyperacute care to secondary prevention, to reduce the risk of adverse CV outcomes [96, 97]. For individuals who are hospitalised in stroke units, diabetes hyperacute management should focus on glycaemic control. Glycaemic control can be pursued with insulin in the hyperacute stage. Tailored care at discharge may be a critical factor to orient individual behaviour and adherence. Hospitalisation can also be a critical opportunity to educate patients and caregivers on the goals of diabetes care and CVD prevention [96, 97].

Stroke programmes include a short-term planned follow-up, with a 3 month evaluation to revise treatment and check clinical status. This may represent a critical occasion to check for glycaemic control and to check treatment adherence, particularly with regard to SGLT2 inhibitors and GLP1-RAs. A three-question strategy for neurologists may be implemented [96] to ensure that the treating clinician is in charge of diabetes treatment, that glycaemic control is at target and that the treatment strategy is optimised for people with stroke and diabetes. Short-term follow-up can also be beneficial for fostering lifestyle changes, including increasing physical activity levels, following a healthy diet and obesity management, as well as quitting smoking [96, 97]. This approach can empower individuals to have direct control of their CV risk profile, and provide endocrinologists with a complete assessment of cerebrovascular status by their neurology peers. Good communication with general practitioners will also enable an excellent network of care to be built around patients, allowing treatment to be tailored depending on their needs.

## Conclusions

Diabetes and stroke are deeply intertwined, with a 3% annual increase in risk of stroke per year of diabetes duration [11]. Microvascular damage can develop before the clinical onset of diabetes and worsen with deteriorating glucose tolerance, supporting early management to lower CV risk [20]. Diabetes and hyperglycaemia are risk factors for poor neurological outcome in ischaemic and haemorrhagic stroke. The management of hyperglycaemia in the hyperacute setting can be directed towards euglycaemia in acute ischaemic stroke, aiming for 7.8–10 mmol/l (140–180 mg/dl) blood glucose in cases of severe hyperglycaemia. In haemorrhagic stroke, intensive glucose control (6.1–7.8 mmol/l [110–140 mg/dl] in those without diabetes and 7.8–10 mmol/l [140–180 mg/dl] in those with diabetes), when applied in the context of a bundle of care including blood pressure, coagulation and temperature control, can have a higher chance of recovery and survival. In terms of CVD prevention, SGLT2 inhibitors and GLP1-RAs should be broadly considered for the treatment of diabetes in individuals with a moderate to high CV risk, with special consideration given to people with AF, HF and CKD. A multidisciplinary approach should be encouraged, with the dual aim of limiting the negative impact of hyperglycaemia in the hyperacute setting and tailoring preventive strategies to people with diabetes in the longer term.

### Supplementary Information

Below is the link to the electronic supplementary material.

Supplementary file1 (PPTX 675 KB)

## Abbreviations

AF: Atrial fibrillation
ASCVD: Atherosclerotic cardiovascular disease
CKD: Chronic kidney disease
CV: Cardiovascular
DPP-4: Dipeptidyl peptidase-4
ESC: European Society of Cardiology
EVT: Endovascular thrombectomy
GLP-1 RA: Glucagon-like peptide-1 receptor agonist
HF: Heart failure
HFpEF: Heart failure with preserved ejection fraction
HFrEF: Heart failure with reduced ejection fraction
ICH: Intracerebral haemorrhage
IVT: i.v. thrombolysis
LV: Left ventricular
LVO: Large vessel occlusion
rt-PA: Recombinant tissue plasminogen activator
SGLT2: Sodium–glucose cotransporter 2
SVD: Small vessel disease
