# Supplementary material for: Prevention and treatment of ischaemic and haemorrhagic stroke in people with diabetes mellitus: a focus on glucose control and comorbidities
Source: Diabetologia. 2024 Apr 16;67(7):1192–205. doi: 10.1007/s00125-024-06146-z (PMC11153285; doi:10.1007/s00125-024-06146-z)
Supplement: Supplementary file 1 — Supplementary file1 (PPTX 675 KB) [file 125_2024_6146_MOESM1_ESM.pptx]

## Slide 1
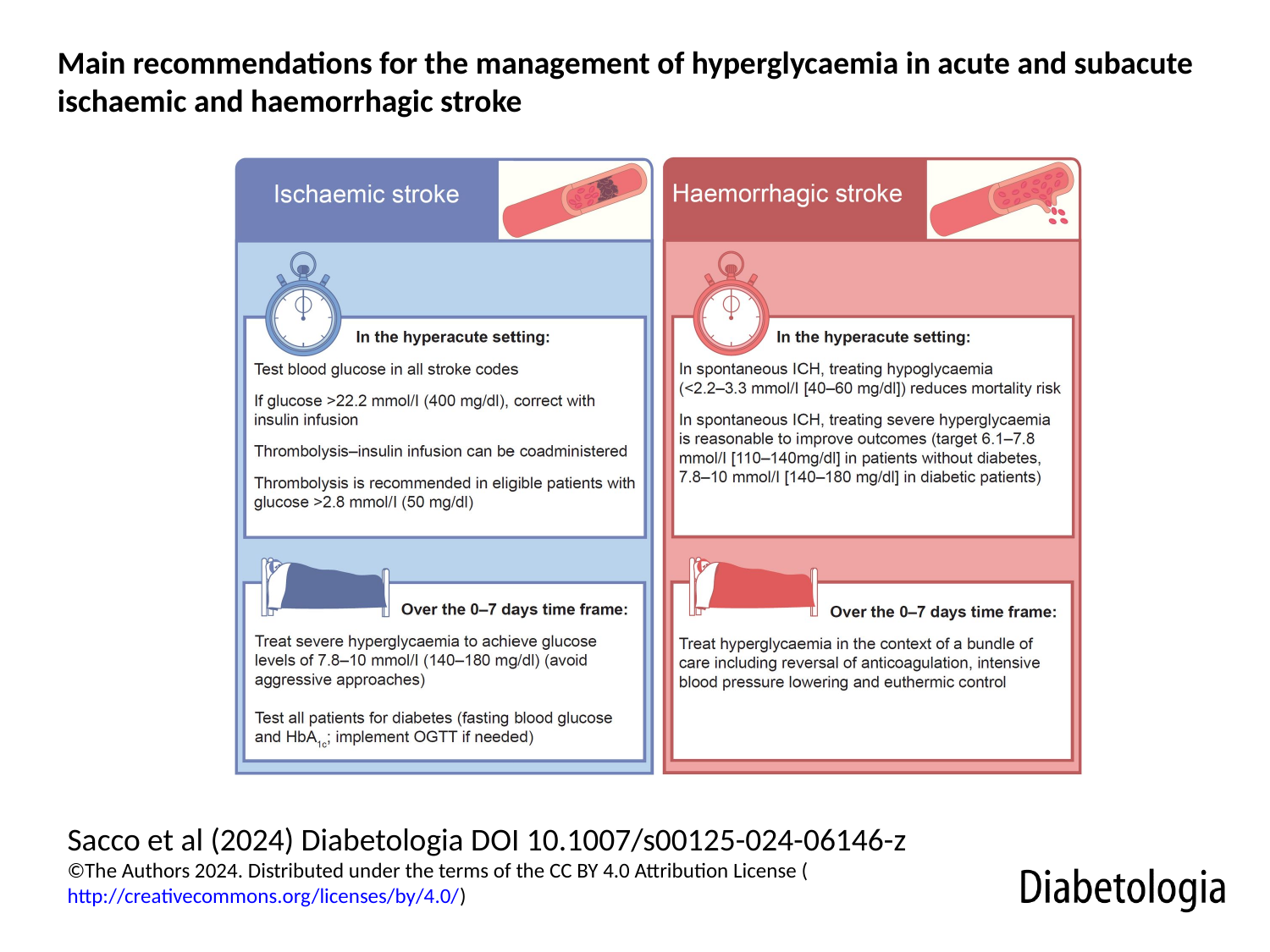

Main recommendations for the management of hyperglycaemia in acute and subacute ischaemic and haemorrhagic stroke
Sacco et al (2024) Diabetologia DOI 10.1007/s00125-024-06146-z
©The Authors 2024. Distributed under the terms of the CC BY 4.0 Attribution License (http://creativecommons.org/licenses/by/4.0/)

## Slide 2
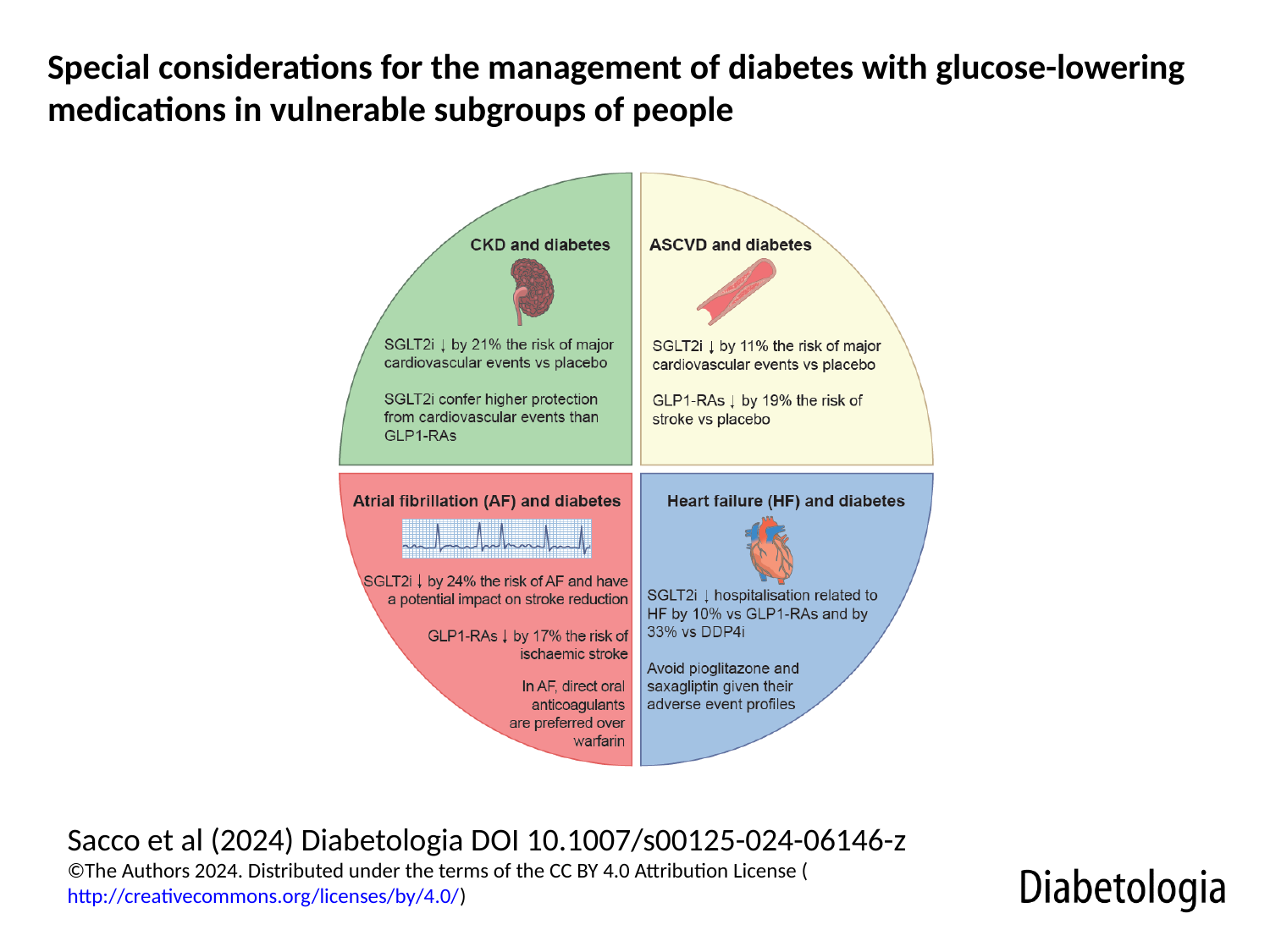

Special considerations for the management of diabetes with glucose-lowering medications in vulnerable subgroups of people
Sacco et al (2024) Diabetologia DOI 10.1007/s00125-024-06146-z
©The Authors 2024. Distributed under the terms of the CC BY 4.0 Attribution License (http://creativecommons.org/licenses/by/4.0/)

## Slide 3
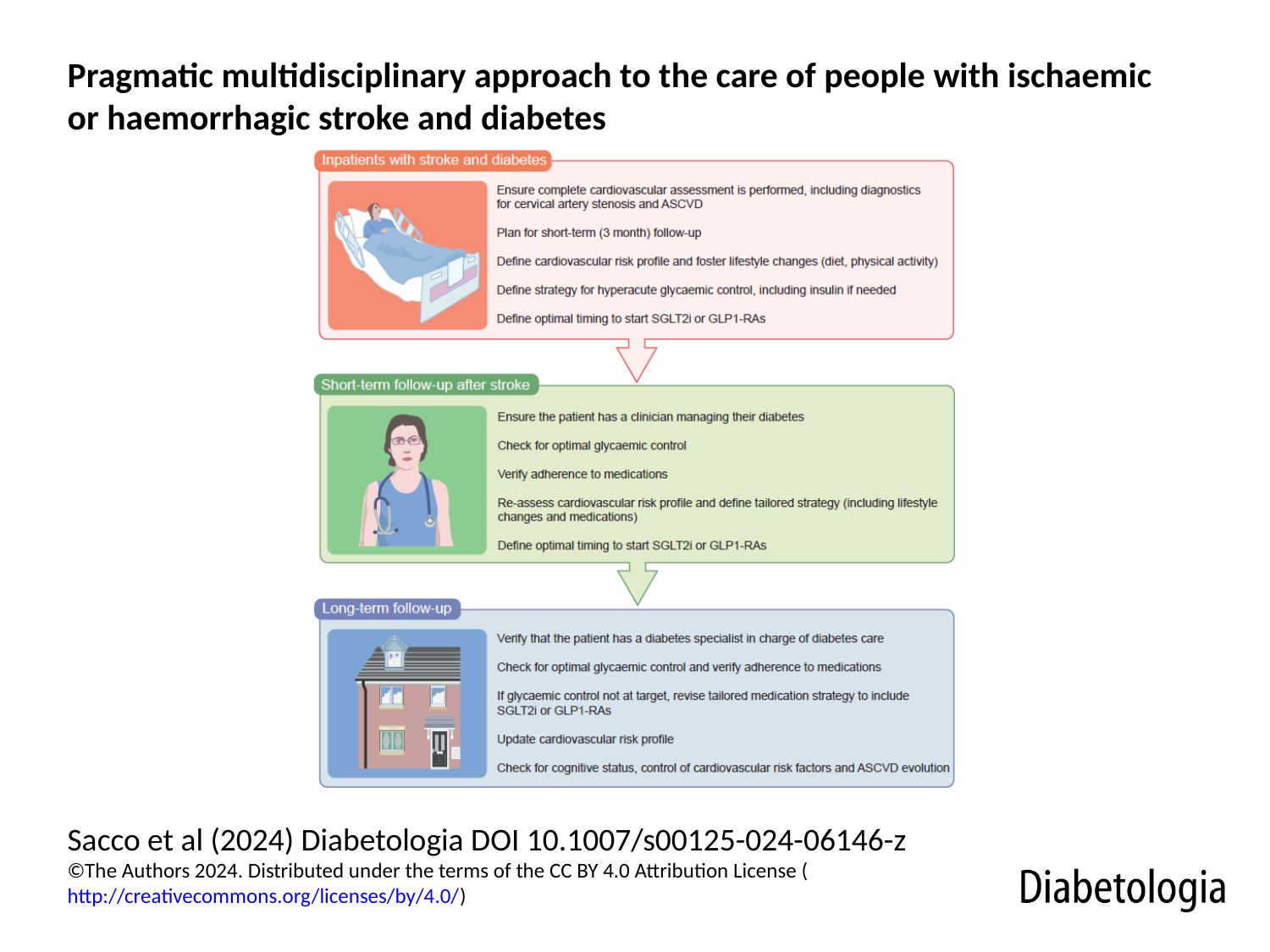

Pragmatic multidisciplinary approach to the care of people with ischaemic or haemorrhagic stroke and diabetes
Sacco et al (2024) Diabetologia DOI 10.1007/s00125-024-06146-z
©The Authors 2024. Distributed under the terms of the CC BY 4.0 Attribution License (http://creativecommons.org/licenses/by/4.0/)
